# Delivery should happen soon and my pain will be reduced: understanding women's perception of good delivery care in India

**DOI:** 10.3402/gha.v6i0.22635

**Published:** 2013-11-22

**Authors:** Sanghita Bhattacharyya, Aradhana Srivastava, Bilal Iqbal Avan

**Affiliations:** 1 Public Health Foundation of India, New Delhi, India; 2 Faculty of Infectious & Tropical Disease, Department of Disease Control, London School of Hygiene & Tropical Medicine, London, UK

**Keywords:** childbirth, delivery care, India, maternal, quality of care, respectful care

## Abstract

**Background:**

Understanding a woman's perspective and her needs during childbirth and addressing them as part of quality-improvement programmes can make delivery care safe, affordable, and respectful. It has been pointed out that the patient's judgement on the quality and goodness of care is indispensible to improving the management of healthcare systems.

**Objective:**

The objective of the study is to understand the aspects of care that women consider important during childbirth.

**Design:**

Individual in-depth interviews (IDIs) and focus-group discussions (FGDs) with women who recently delivered were the techniques used. Seventeen IDIs and four FGDs were conducted in Jharkhand state in east India between January and March 2012. Women who had normal deliveries with live births at home and in primary health centres were included. To minimise recall bias, interviews were conducted within 42 days of childbirth. Using the transcripts of interviews, the data were analysed thematically.

**Results:**

Aspects of care most commonly cited by women to be important were: availability of health providers and appropriate medical care (primarily drugs) in case of complications; emotional support; privacy; clean place after delivery; availability of transport to reach the institution; monetary incentives that exceed expenses; and prompt care. Other factors included kind interpersonal behaviour, cognitive support, faith in the provider's competence, and overall cleanliness of the facility and delivery room.

**Conclusions:**

Respondents belonging to low socio-economic strata with basic literacy levels might not understand appropriate clinical aspects of care, but they want care that is affordable and accessible, along with privacy and emotional support during delivery. The study highlighted that healthcare quality-improvement programmes in India need to include non-clinical aspects of care as women want to be treated humanely during delivery – they desire respectful treatment, privacy, and emotional support. Further research into maternal satisfaction could be made more policy relevant by assessing the relative strength of various factors in influencing maternal satisfaction; this could help in prioritising appropriate interventions for improved quality of care (QoC).

India accounts for 19% (56,000) of annual global maternal deaths (1). Under the current health sector reform programme in India, implemented through the National Rural Health Mission (NRHM) since 2005, strategies for maternal and new-born health include provision of quality antenatal care, ensuring access to a skilled birth attendant, institutional and home-based new-born care and referral linkages. In this context, studies on women's perception of care received during childbirth are very relevant given the government's accelerated efforts towards increasing institutional deliveries and improving quality of services with the long-term aim of bringing about appreciable reduction in maternal mortality (2, 3). Such evidence would help determine aspects of care that women value most during childbirth to support demand-generation and remove barriers in service delivery.

In developing-country contexts, with considerable gaps in services, emphasis has been placed on increasing service availability, but efforts to ensure quality of care (QoC) and recording women's perceptions and needs during childbirth have not been sufficient (4, 5). However, interest in the quality of health services in developing countries is growing, with increasing efforts towards maintaining acceptable quality standards (6). The concept of quality broadly encompasses clinical effectiveness, safety, and a good experience for the woman and implies care that is effective, patient-centred, timely, efficient, and equitable (7, 8). Maternal satisfaction is a related concept, aiming to determine ‘individual perceptions of the quality of health care delivered’ (9). It is the ‘patient's judgement on the quality and goodness of care’ and is essential to improved design and management of healthcare systems (10–12). This is also a process of democratisation of health services, as service is then oriented to meet users’ expectations (13).

There are few studies on user perception and satisfaction with services in India and those that have been conducted are largely restricted to family planning (14). Some studies explored the reasons for women's choice of place of delivery, home versus institutional delivery and public versus private facilities (15–17). Others focus on women's choices and experience in tertiary care facilities (18, 19). However, evidence to understand the type of care a woman needs during time of delivery, irrespective of place of delivery, is limited.

Against this background, there is a need for evidence to understand a woman's perspective of care during delivery since if what she considers as important receives attention, this can lead to maternal satisfaction and further uptake of service. Consequently, it will also enhance the effectiveness of the programme in achieving positive outcomes in terms of reduced maternal and neonatal mortality. The objective of this study is to understand the aspects of care women consider important during childbirth.

## Methods

The study was conducted between January and March 2012 and comprised in-depth interviews (IDIs) and focus-group discussions (FGDs) with women who had delivered in the 42 days prior to the study.

### Study area

The study was undertaken in Jamtara district in Jharkhand, a state in eastern India with poor maternal and child health indicators (20). In spite of the conditional cash incentive scheme to promote institutional deliveries, institutional deliveries account for just 40% of the total deliveries in the state, well below the national average of 73% (3).

### Participants

The study population was limited to women who had delivered within 42 days prior to the interview to minimise recall bias. Only women who had had a normal delivery and live birth were interviewed, since women who had complicated deliveries and stillbirths would have different perspectives of care. The villages in the study district were randomly selected and the list of women who had delivered in the last 42 days prior to the study from those selected villages was obtained from the community health worker. Based on the listing, the research team identified women who met the criteria, that is, who had normal deliveries and live births. Women were also segregated by their place of delivery (whether in institutions or at home) and the sample had almost equal representation of both. For the IDIs, the research team approached every third eligible woman from the list, explained the purpose of the study, and sought her verbal consent for the interview and recording. There were five refusals; two on account of ill health of the respondent and three on account of non-availability of time for the interview. While the criteria for selecting FGD participants were the same as the IDIs, it was ensured that respondents participating in FGDs were different from those who participated in IDIs. Four FGDs were conducted. Each FGD had 7–9 participants and constituted two groups of women who had had home births and two groups of women who had delivered at an institution. A total of 49 respondents participated in the 17 IDIs and 4 FGDs.

### Study instruments

Semi-structured guides which included open-ended questions were used to conduct the interviews and FGDs. The guides were pre-tested and peer-reviewed by national-level maternal health experts and representatives of local non-governmental organisations. They were translated into the local languages (Hindi and Bengali) used in the study area.

The study was carried out in two phases: first, the IDIs were conducted and FGDs were then conducted to further validate the responses. The IDI guides were developed on the basis of a long list of factors identified from a review of literature on women's perceptions and satisfaction with maternal care in developing countries. The researcher adequately probed for responses on all the aspects of care listed in the IDI guide. The factors included were promptness of services such as availability of the health provider and transport to reach the institution; provision of appropriate medical care (primarily medicine); emotional support and privacy provided during delivery; cleanliness and hygiene of the place of delivery; kind interpersonal behaviour, cognitive support, and faith in the provider's competence. By narrating the story of her delivery process, the woman could recall her experience and explain the aspects of care she considered important during childbirth. A preliminary analysis of the IDI transcripts was carried out to understand the commonly cited factors that women considered important. The FGD guide was developed to investigate women's perspectives on those specific factors. This was done to understand in detail the importance of the factors that emerged as critical to women's experience of care. Also during FGDs, the facilitator encouraged participants to discuss new factors that they felt were missing. It was also expected that in a group, women would be more forthcoming in narrating their experiences, particularly regarding any shortfall in service delivery by health facility staff (such as the issues of abuse and informal payments at facilities).

### Data collection and management

IDIs with women were conducted at their residences and FGDs were held in common meeting places in the villages. Interviewing women in the health facility or in the presence of any service providers was avoided, as it could lead to bias in reporting the experience of childbirth. Each interview lasted between 40 and 90 min. At the beginning, the participants had the general topics explained to them and were encouraged to express their ideas freely. Care was taken to ensure that all themes of care were covered in the discussion. Other relevant issues that emerged were also encouraged.

Two researchers with experience in qualitative research and knowledge of local dialect of the area conducted the interviews. One researcher conducted the interviews and the other noted important discussion points during the interviews. The researcher who was most familiar with the local dialect was the primary facilitator of the FGDs. At the end of each interview, the researchers completed the section on notes and observations. This was intended to capture information on the ease of the interview, whether respondents answered questions freely, and any other relevant information. At the end of each day, the researchers reflected upon and discussed the interviews conducted to improve the way in which subsequent interviews were conducted. A voice recorder tape was used to record the sessions and an independent researcher transcribed the interviews in the local dialect. The transcripts were then translated into English.

### Data analysis

Transcripts were analysed using the Atlas TI software, using a thematic analytical approach. Responses were categorised on the basis of *a priori* codes or themes identified from the literature review on determinants of maternal satisfaction with care in developing countries. Other themes emerging from the transcripts were also identified and included in the discussion.

### Ethical consideration

The Public Health Foundation of India (PHFI) Institutional Ethics Committee (IEC) and the University of Aberdeen (Aberdeen, United Kingdom) approved the research. At the time of interview, the informed consent form was read and explained to respondents. As most of the respondents in the study area were illiterate, a verbal consent was taken that was recorded prior to the interview. Confidentiality was assured during the data analysis and quotes were given with pseudonyms.

## Results

### Characteristics of women

Most respondents were 18–23 years old and illiterate; only a few had education to primary level. They were mostly Hindus and belonged to a lower social class. The majority belonged to low-income groups with their husbands working as casual labourers. Parity was from 2 to 4 children and the outcome of pregnancy in all cases was live birth. Details are provided in Table 1.

**Table 1:** Demographic profile of the respondents

	Participants in in-depth interviews *N*=17	Participants in focus-group discussion *N*=32
Age		
18–20	5	18
21–25	12	18
Educational status		
Illiterate	14	14
Literate, informal education	2	13
Primary level (1–4 class)	1	5
Religion		
Hindu	11	23
Muslim	6	9
Caste		
General	4	0
Scheduled caste	9	22
Scheduled tribe	4	10
Main economic activity of the head of the household		
Cultivator	2	3
Agricultural labourer	4	4
Casual labourer	11	18
Others	0	7
Total number of children		
1	7	6
2–3	9	19
More than three children	1	7

### Women's perception of good delivery care

Women narrated their experience during delivery and were asked about what aspects of care they valued most during the time of delivery. The most common responses were: availability of health providers and appropriate medical care (primarily medicine) in case of complications; emotional support; privacy during delivery; clean place after delivery, availability of transport to reach the institution; monetary incentives that exceeded expenses and prompt care (Table 2). Other valued aspects of care included kind interpersonal behaviour, cognitive support, faith in provider's competence, and overall cleanliness of the facility and delivery room.

**Table 2:** Women's perception of good delivery care

	Women's perception of good delivery care	
	
Key dimensions of care*	Commonly cited aspects	Other aspects of care
**Structure** The resources in terms of infrastructure, equipment, drugs and supplies to provide quality care and also the care provided by appropriately trained and supervised providers	Availability of provider Availability of medicine in case of complication and pain management Availability of transport to access health facility	
**Process** Care consistent with scientific knowledge, internationally recognised good practice and that is safe (clean birth practices, avoidance of iatrogenic harm); timely and responsive (respectful, promoting autonomy, equitable).	Emotional support: family members present during delivery Privacy: delivery in a secluded place and absence of male members Hygiene of the delivery place particularly cleanliness of the delivery place The cost of the services Promptness in care	Cognitive support: information provided by provider Kind interpersonal behaviour with the provider Perceived provider competence Perceived completeness of procedures Cleanliness of facility and labour room
**Outcome** Good clinical outcome (e.g. mortality reduction)	–	_
**Other aspects**		Influence of community health workers and peers in deciding the place of delivery

* Based on Refs. 8, 12, and 25.

The commonly cited aspects of care are discussed in detail below.

#### Availability of health providers and appropriate medical care

Women considered the presence and availability of trained medical personnel and supplies in the form of medicines as a critical aspect of care and an important reason for preferring institutional delivery. For this reason, some women previously had home births said they would prefer to have institutional delivery for the next child, as doctors and nurses are in a better position to assess the condition of the child and mother. Women felt more secure at the facility as there was the assurance of appropriate medical care in case of any emergency.

Although clinically not recommended, women wanted drugs to hasten the labour process. Even if these had to be purchased from a pharmacy, women did not complain as they considered the drugs to be crucial for delivery care. Women with home births also usually called a private doctor to provide pain-relieving or labour-inducing medication before delivery. Non-availability of medicine and injections was one of the main reasons women felt it was not safe to have home births in case of any complications. They knew that the traditional birth attendant (TBA), referred to in India as ‘Dai’ who conducts delivery at home does not have any medicine and therefore is unable to administer injections to induce labour.

*Availability of transport* is one of the major reasons for influencing place of delivery. Inability to arrange transport is a key reason for home deliveries particularly for deliveries that occur during the night. Despite health facilities in this study being geographically accessible, women reported they could not reach the institution due to non-availability of transport at the time of labour. Difficulty in communication and arrangement of transport is one of the key factors determining most of the home births. Most women reported that by the time the community health worker could arrange transport, they had already delivered.

...but what to do. When my labour pain started it was midnight, there was no facility of vehicle and we did not have enough money to hire a vehicle. – Rani, who delivered at home.

Accessibility to the institution by the provision of a readily available vehicle is a key issue for women in terms of defining what constitutes good care.

#### Emotional support

The presence of family members is one of the key aspects that women believe constitutes good care, whether she is delivering at home or at an institution. According to traditional culture, generally a female family member, either mother or mother-in-law, accompanies the woman during child birth. In the institution, the community health worker is also often present in the delivery room along with the woman's family members, which is highly appreciated since this provides a lot of emotional support in an unknown hospital environment. Women who had delivered at home feared the unknown environment of a facility, particularly the presence of male doctors. For women delivering at home, the presence of family members and Dai provided emotional support. In most cases, the Dai belonged to the same neighbourhood, and for many women she had assisted in their previous delivery also.

At home all the family members are present and all the worries and tension were automatically gone. – Sita who delivered at home.

*Privacy during delivery* is highly valued, whether women delivered at home or in institutions. Delivering in a secluded place with no male presence is most desirable. The presence of male staff is one of the reasons for women not willing to deliver at the institution.

I am scared that anybody can touch me and also feel that men will also be there during the delivery. – Padma, who delivered at an institution.

Although many women complained that the place was a bit crowded, the crowd was limited to other pregnant women and the community health worker accompanying them. The internal check-up before delivery was done by an auxiliary nurse midwife (ANM) in a separate room where no male doctors or staffs were present, and women felt neither uncomfortable nor embarrassed.

No, I didn't feel like that (embarrassed), because there were only women there (in the check-up room). – Namita, who delivered at a health facility.

Women who had delivered at home stated that there was no separate room for delivery but as childbirth is considered to be a feminine process, only female members of a family and neighbours were present in the room at time of delivery. This made women feel more comfortable at home. One woman, however, reported that she had delivered the baby in the presence of her older male child, as there was no other place for him to go. Even then she was comfortable but she felt this was inevitable due to scarcity of rooms in the house.

The men were not at home, they had gone for work... and anyways, had they been at home, they would have definitely gone out... even they understand this and after giving injection the doctor was supposed to go out from the room. – Najma, who delivered at home.

#### Hygiene of the place after delivery

Another aspect of delivery care that was very important to these women was the issue of cleanliness, both of the place and the cleaning of her new-born. After the birth at an institution, the staff who also clean the delivery place cleaned the child and the mother. Women perceived this as a major advantage, because at home though the Dai cleans the delivery place, cleaning is considered a hassle as it involves re-coating the floor with a fresh layer of mud.

*Expenditure* is another deciding factor for determining good care and a reason behind choosing the place of delivery. According to some respondents, it was more expensive to deliver at home than at the institution, where certain costs, such as buying medicines, paying doctors’ fees, and some of the Dai costs are taken care of. However, women still have to spend quite a bit of money at the hospital, mainly in the form of tips to nurses and Dais, and sometimes for buying certain medicines/injections. The birth of a child is an occasion of happiness for the family, and the family distributes sweets to friends and relatives to share the happiness. This custom has paved the route for a lot of informal expenses for the family in the form of tips to the providers.

... I gave 600 rupees to the nurse, who assisted in the delivery. Community health worker was also asking for rupees 100... – Pooja, who delivered at a health facility.

Regardless of such expenses, women perceive institutional delivery to be monetarily more beneficial, particularly after the introduction of the conditional cash transfer (CCT) called Janani Surakshya Yojna, where the government pays a woman INR 1,400 ($25) after she delivers at a health facility. Though there is a time lag between the birth of the child and when they get money from the CCT scheme, women reported they felt more secure incurring such expenses in the knowledge that it would be covered by the money that they would receive from the government.

We are poor people, so if the government opens up free facilities, we will go. If there are facilities provide eating-drinking, medicines, and doctors are available. We will go there. – Payel, who delivered at an institution.

#### Promptness in care

Almost all of the women were satisfied with the promptness of services as they were attended to soon after their labour pain had started. Those who had delivered at a health facility reported that there was minimum waiting time between their arrival to the facility, getting admitted, and being monitored by health personnel. Promptness of care is a key criteria of perceived good care.

I just feel that the delivery should happen as soon as possible and my pain is reduced. That's all that I wanted.

This quote from a woman with a low literacy level, who cannot coherently express good clinical care, sums up the care she desires during childbirth. This encompasses readily available transport, availability of a skilled birth attendant, and appropiate care to make the whole delivery process a better experience.

## Discussion

The study provides an insight into women's experience during childbirth and the factors that are most important to them during delivery. The perception of good care that emerged from the present study further complements the factors that have emerged from similar studies in developing countries (21–24). Factors such as emotional support, promptness in care, privacy, accessibility to the health facility, availability of skilled health providers, appropriate medical care, and cost of services are reported to be important aspects of care. Women in this study belonging to low socio-economic strata and with a basic literacy level might not understand clinical aspects of care, but they want care that is affordable and accessible, along with privacy and emotional support during delivery, irrespective of whether they deliver at home or at a health facility.

QoC encompasses dimensions of structure, process, and outcome of care, and to achieve a holistic improvement in QoC each aspect needs to be given equal emphasis as part of any quality-improvement initiative (8, 12, 25). The recent quality-improvement initiatives in India have emphasised improvement of infrastructure, human resources, supplies, and equipment. However, the second domain of quality, that is, process of care which includes, promptness, responsiveness, and women-centred behaviours, such as respect, dignity, and emotional support have not been given due attention. A review of the implementation of Reproductive and Child Health programme in five states in India focused on structural and management indicators, with very little coverage of processes of care (26).

The study findings highlight that along with structural aspects of quality, the process of care, which includes emotional support, privacy during childbirth, and promptness of care is equally important for women during childbirth. Expenditure is also one of the deciding factors for maternal satisfaction with care (23, 24). The study highlighted that due to the conditional cash incentive scheme in India, the community health workers motivate women to opt for institutional delivery as they are assured of free delivery care along with monetary incentives. However, the issue of expenses such as informal payments at facilities and purchasing medicines from private providers might deter women in accessing the service in the future. Such aspects of care a woman can recognise and appreciate easily and these experiences can have a profound influence on acceptance, uptake, and use of services (27–31). Simultaneously, structural elements of quality like publicly accessible transport, availability of providers, and essential medicines are also major factors in ensuring that women choose to go to facilities for delivery (24, 32).

### Limitations

The study may not be a true representation of the entire population in India as it is based on a purposive small sample. Moreover, the sample comprises women in a younger age group, with basic educational levels who belong to a lower economic status, all of which can have an implication on their perception of good care during delivery.

## Conclusion

This study adds to the increasing recognition of the importance of understanding women's perspectives and their needs during childbirth. The study highlighted that healthcare quality-improvement programmes in India also need to address non-clinical aspects of care as women want to be treated humanely during delivery – they desire respectful treatment, privacy, and emotional support. It can consider the birth companion of the woman's choice and support from family members during delivery at a health facility. It also highlights the importance of raising awareness among providers about respectful attitudes and behaviour towards women. If a woman is not confident about receiving the same level of comfort during delivery at a health facility in terms of emotional support and privacy that she would get at home, she might be dissuaded from seeking an institutional birth. Further research could be made more policy relevant by assessing the relative strength of various factors in influencing maternal satisfaction; this could help in prioritising appropriate interventions for improved QoC. Understanding women's perceptions of good care and addressing them in quality-assurance programmes can not only bridge the supply and demand gap but can also increase facility-based delivery by assuring safe, affordable, and respectful care.
